# Estimating length of stay and inpatient charges attributable to hospital-acquired bloodstream infections

**DOI:** 10.1186/s13756-020-00796-5

**Published:** 2020-08-18

**Authors:** Yuzheng Zhang, Mingmei Du, Janice Mary Johnston, Ellie Bostwick Andres, Jijiang Suo, Hongwu Yao, Rui Huo, Yunxi Liu, Qiang Fu

**Affiliations:** 1 School of Public Health, The University of Hong Kong, Patrick Manson Building, 7 Sassoon Road, Pokfulam, Hong Kong, China; 2 Department of Infection Management and Disease Control, Chinese PLA General Hospital, No. 28 Fuxing Road, Haidian District, Beijing, China; 3 XingLin Information Technology Company, No. 57 Jianger Road, Binjiang District, Zhejiang, Hangzhou China; 4 China National Health Development Research Center, No.9 Chegongzhuang Street, Xicheng District, Beijing, China; 5 National Center for Healthcare Associated Infection Prevention and Control, Beijing, China

**Keywords:** Hospital-acquired bloodstream infection, Length of stay, Hospital charge

## Abstract

**Background:**

Hospital-acquired bloodstream infection (BSI) is associated with high morbidity and mortality and increases patients’ length of stay (LOS) and hospital charges. Our goals were to calculate LOS and charges attributable to BSI and compare results among different models.

**Methods:**

A retrospective observational cohort study was conducted in 2017 in a large general hospital, in Beijing. Using patient-level data, we compared the attributable LOS and charges of BSI with three models: 1) conventional non-matching, 2) propensity score matching controlling for the impact of potential confounding variables, and 3) risk set matching controlling for time-varying covariates and matching based on propensity score and infection time.

**Results:**

The study included 118,600 patient admissions, 557 (0.47%) with BSI. Six hundred fourteen microorganisms were cultured from patients with BSI. *Escherichia coli* was the most common bacteria (106, 17.26%). Among multi-drug resistant bacteria, carbapenem-resistant *Acinetobacter baumannii* (CRAB) was the most common (42, 38.53%). In the conventional non-matching model, the excess LOS and charges associated with BSI were 25.06 days (*P* < 0.05) and US$22041.73 (*P* < 0.05), respectively. After matching, the mean LOS and charges attributable to BSI both decreased. When infection time was incorporated into the risk set matching model, the excess LOS and charges were 16.86 days (*P* < 0.05) and US$15909.21 (*P* < 0.05), respectively.

**Conclusion:**

This is the first study to consider time-dependent bias in estimating excess LOS and charges attributable to BSI in a Chinese hospital setting. We found matching on infection time can reduce bias.

## Background

Bloodstream infection (BSI) is a serious adverse event associated with high morbidity and mortality. Studies in Canada demonstrated that 28% of BSIs are nosocomial [1], and data from North America and Europe showed BSI ranks among the top seven causes of death [2]. Our previous study in a Chinese tertiary hospital indicated an increasing incidence of BSI from 0.53 to 0.65 per 1000 patient-days over 5 years (2013–2017) [3]. BSIs are also associated with increased length of stay (LOS) and medical costs [4–6]. However, infection control programs are often regarded as cost centers and potential areas for budget cuts rather than revenue generators [7]. In fact, reduced healthcare associated infections (HAI) and LOS could increase the bed turnover rate and hospital revenue [8]. Appropriate analysis methods can help decision-makers understand the clinical and economic burden of BSIs and choose the most cost-effective infection control strategies.

Estimations of BSI hospital related costs vary widely. Most studies do not account for the time-varying nature of HAIs, thereby overestimating the cost of BSI episodes [9, 10]. In conventional methods, the time spent in the hospital prior to the occurrence of the HAI is incorrectly attributed to the HAI, thus inflating attributable LOS and cost [11]. Two methods have been used to overcome the overestimation problem: multistate model and matching on infection time [11]. Multistate models treat the infection as one of several mutually exclusive states (e.g. discharge/death) after a patient’s admission. The method of matching on infection time (risk-set matching) matches each BSI case with a comparable control patient at risk of infection at the cases’ infection time [12].

Variance in health insurance and reimbursement systems also impact attributable LOS and cost of BSI. Currently, there are few economic studies about HAI in Mainland China. Studies from Hubei and Sichuan demonstrated that the attributable cost of HAI were US$6173.02 and $2439.77 per case, respectively [13, 14]. Another Chinese study of catheter-related bloodstream infections indicated the total cost attributable was US $3528.6 per case [15]. However, these HAI cost studies in Mainland China did not account for disease severity or time-dependent bias. Hence, our primary objective is to estimate the LOS and hospital charges attributable to BSI with time-varying exposures, and to compare our results to those obtained using conventional methods to understand the magnitude of the time-dependent biases.

## Methods

### Data sources

A retrospective observational cohort study was conducted at a tertiary hospital with 3800 beds in Beijing. The hospital conducted hospital-wide HAI surveillance with a real-time nosocomial infection surveillance system (RT-NISS). The study included patients admitted and discharged between January 1st and December 31st 2017. We excluded patients in outpatient settings, physical examination centers, and day surgery centers. There was no age limit. Data was divided between two groups: (1) cases, including patients with nosocomial bloodstream infections and (2) controls, comprised of patients without BSIs (or other HAIs). To protect patient privacy, the study excluded sensitive patient identifiers (e.g. name and identification numbers). Ethical approval (number: S2019–142-02) was obtained from the Medical Ethical Committee of the Chinese PLA General Hospital.

### Case definition

BSIs were identified should meet the following criteria: (1) isolation of bacteria from at least one blood culture, (2) exclusion of contaminated blood samples during the collection and culture, (3) one of the following clinical symptoms: fever (> 38 °C), chills, or hypotension. Only one blood culture positive of the common skin commensals organisms (e.g. coagulase-negative staphylococcus [CoNS], non-*diphtheriae Corynebacterium* spp., *Bacillus* spp., *Propionibacterium* spp., viridans group streptococci, *Aerococcus* spp., and *Micrococcus* spp.) were excluded [16]. Based on the BSI criteria, hospital-acquired BSIs were defined as the first positive blood culture obtained≥48 h after hospital admission and with no evidence of infection at admission. Time of infection was defined as the day of physician confirmation, which generally corresponded with the collection time of first positive blood sample.

### Microbiological test

Blood was cultured with BacT/ALERT 3D system (Becton-Dickinson, Sparks, MD, USA). Microorganism species were identified using the VITEK 2 system (BioMérieux, Marcy 1 Étoile, France). Antibiotic susceptibility testing was determined by the VITEK 2 system or the Kirby-Bauer Disk Diffusion method (Oxford, UK) in accordance with the guideline proposed by the Clinical and Laboratory Standards Institute (CLSI). According to the World Health Organization (WHO) priority list of antibiotic-resistant bacteria, the study included corresponding antibiotic-resistant bacteria responsible for BSI, such as: carbapenem-resistant *Acinetobacter baumannii*, carbapenem-resistant *Pseudomonas aeruginosa* [17].

### Variables of interest

Demographic information (age, sex, region, insurance type), diagnosis code (based on ICD-10), intensive care unit (ICU), and procedure-related information (receiving surgery, central line, urinary catheter, ventilator) were collected. The Charlson comorbidity index was calculated for each patient to capture the severity of comorbidities [18].

The outcomes of interest were LOS and inpatient charges. Hospital charges are the hospital fees for services, medicine, and materials, etc. The patients’ individual hospital charges were retrieved from the hospital information system. Mean charge was used for two patients with missing hospital charge data. Medical charges were collected in the Chinese currency Renminbi and converted into US dollars ($) according to the exchange rate (1USD = 6.53 RMB) issued by the Bank of China on 31 December 2017.

### Statistical analysis

We conducted the analyses with three different models. Model 1, conventional non-matching, compares the BSI patients’ charges and LOS with those of patients without BSI. This method ignores confounders and the time-dependent nature of costs and LOS.

Model 2, conventional propensity score matching (PSM), estimates the propensity scores with logistic regression based on the variables listed in Table 2. The matching variables (i.e. status of central line, urinary catheter, and ventilator before infection) include only information available at time of admission (or within the first 2 days). Instead of exact matching the BSI and non-BSI group on the dependent variables, the PSM matches by the propensity score at a 1:1 ratio. We used nearest-neighbor matching with a caliper width of 0.25.

Model 3, risk-set matching, matches patients who experienced a HAI on a specific day to similar patients who have not yet experienced HAIs at that point in their hospital admission. We estimated the risk-set propensity score using Cox proportional hazards regression. The survival outcome was either BSI (i.e., an event) occurring and its corresponding time or BSI not occurring (i.e., a censored event) and the length of stay in the hospital (i.e., censoring time). The same dependent variables included in Model 2 were used in Model 3, except for some time-varying covariates. These variables (receiving central line, urinary catheter, ventilator) were recoded weekly. The risk-set propensity scores were estimated by linear prediction with the time-varying Cox regression model, which was described in previous studies [12, 19]. A nearest-neighbor matching was applied with the risk-set propensity score. Each BSI case infected at time T_1_ is matched to a patient not yet infected at time T_1_ rather than a never-infected patient. The matched patient in the control group may acquire an infection later than T_1_, in which case they would be classified in both the infected and uninfected group.

The degree of balance between the matched pairs were evaluated using standardized mean differences (SMD). The cutoff for interpreting the magnitude of SMD was defined as followings: small, 0.2; medium, 0.5; and large, 0.8 [20]. Continuous and categorical variables were compared between BSI and non-BSI groups using t-test and Chi-square test, respectively. Moreover, a sensitivity analysis was conducted with defining the infection time 2 days before the original infection date to account for a 48-h incubation period. Statistical analyses were performed using R version 3.4.3.

## Results

### Patients and matching

In total, 118,600 patient admissions were included in the study and 557 (0.47%) BSI were identified. Patient characteristics are presented in Table 1. Mean age of all patients was 51.12 years and 53.88% were male. The majority of patients (60.79%) were from northern China (Beijing, Tianjin, Shanxi, Hebei, Inner Mongolia). 33,580 (28.31%) patients had public insurance, 77.19% of which were from Beijing. The remaining 22.81% patients with public insurance cannot claim medical fees from the government since they do not reside in Beijing (public insurance is tied to resident location).

**Table 1:** Patient Characteristics before and after matching between the case and control groups, 2017

	BSI (***N*** = 557)	Model 1		Model 2		Model 3	
		No BSI (***N*** = 118,043)	SMD^*^	No BSI (***N*** = 557)	SMD^*^	Control (***N*** = 557)	SMD^*^
Age (mean, SD)	57.02 (21.3)	51.09 (18.4)	0.297	56.75 (18.1)	0.013	56.29 (17.82)	0.036
Sex, female (n, %)	195 (35.0)	54,505 (46.2)	0.229	206 (37.0)	0.041	224 (40.2)	0.108
Insurance, yes (n, %)	164 (29.4)	33,386 (28.3)	0.026	157 (28.2)	0.028	126 (29.3)	0.156
Region (n, %)			0.161		0.111		0.078
Northeast	52 (9.3)	13,192 (11.2)		67 (12.0)		47 (8.4)	
Eastern	80 (14.4)	18,091 (15.3)		86 (15.4)		77 (13.8)	
Northern	348 (62.5)	71,757 (60.8)		337 (60.5)		356 (63.9)	
Central	30 (5.4)	8387 (7.1)		23 (4.1)		32 (5.7)	
Southern	9 (1.6)	598 (0.5)		9 (1.6)		5 (0.9)	
Southwest	10 (1.8)	1698 (1.4)		8 (1.4)		10 (1.8)	
Northwest	28 (5.0)	4320 (3.7)		27 (4.8)		30 (5.4)	
Charlson score (mean, SD)	2.2 (2.6)	1.6 (2.7)	0.216	2.4 (2.9)	0.066	2.4 (2.9)	0.076
ICU (n, %)	91 (16.2)	4577 (3.9)	0.422	84 (15.3)	0.030	79 (16.9)	0.060
Receive any procedures							
Surgery (n, %)	153 (27.5)	41,014 (34.7)	0.158	155 (27.8)	0.008	126 (22.6)	0.112
Central line catheter (n, %)	246 (44.2)	10,207 (8.6)	0.880	253 (45.4)	0.025	213 (38.2)	0.121
Urinary catheter (n, %)	235 (42.2)	29,255 (24.8)	0.375	220 (39.5)	0.055	213 (38.2)	0.081
Ventilator (n, %)	132 (23.7)	4328 (3.7)	0.609	136 (24.4)	0.017	92 (16.5)	0.180
Primary discharge diagnosis (ICD-10) (n, %)			0.665		0.175		0.259
A00-B99	16 (2.9)	796 (0.7)		15 (2.7)		14 (2.5)	
C00-D48	126 (22.6)	24,090 (20.4)		145 (26.0)		130 (23.3)	
D50-D89	5 (0.9)	384 (0.3)		5 (0.9)		3 (0.5)	
E00-E90	1 (0.2)	2910 (2.5)		0		2 (0.4)	
F00-F99	0	194 (0.2)		0		0	
G00-G99	13 (2.3)	2331 (2.0)		12 (2.2)		11 (2.0)	
H00-H59	0	3146 (2.7)		0		0	
H60-H95	0	2290 (1.9)		0		0	
I00-I99	54 (9.7)	15,562 (13.2)		49 (8.8)		37 (6.6)	
J00-J99	42 (7.5)	3118 (2.6)		37 (6.6)		25 (4.5)	
K00-K93	80 (14.4)	7673 (6.5)		81 (14.5)		84 (15.1)	
L00-L99	1 (0.2)	860 (0.7)		0		1 (0.2)	
M00-M99	15 (2.7)	8178 (6.9)		10 (1.8)		8 (1.4)	
N00-N99	25 (4.5)	7336 (6.2)		18 (3.2)		25 (4.5)	
O00-O99	3 (0.5)	2841 (2.4)		1 (0.2)		5 (0.9)	
P00-P96	6 (1.1)	656 (0.6)		8 (1.4)		8 (1.4)	
Q00-Q99	4 (0.7)	1760 (1.5)		6 (1.1)		3 (0.5)	
R00-R99	5 (0.9)	729 (0.6)		5 (0.9)		6 (1.1)	
S00-T98	34 (6.1)	3265 (2.8)		29 (5.2)		27 (4.8)	
Z00-Z99	127 (22.8)	29,913 (25.3)		136 (24.4)		168 (30.2)	

Model 1, no matching; Model 2, conventional propensity score matching; Model 3, risk set matching. *BSI* Bloodstream infection, *SMD* Standardized mean difference. *A00-B99* Certain infectious and parasitic diseases, *C00-D48* Neoplasms, *D50-D89* Diseases of the blood and blood-forming organs and certain disorders involving the immune mechanism, *E00-E90* Endocrine, nutritional and metabolic diseases, *F00-F99* Mental and behavioral disorders, *G00-G99* Diseases of the nervous system, *H00-H59* Diseases of the eye and adnexa, *H60-H95* Diseases of the ear and mastoid process, *I00-I99* Diseases of the circulatory system, *J00-J99* Diseases of the respiratory system, *K00-K93* Diseases of the digestive system, *L00-L99* Diseases of the skin and subcutaneous tissue, *M00-M99* Diseases of the musculoskeletal system and connective tissue, *N00-N99* Diseases of the genitourinary system, *O00-O99* Pregnancy, childbirth and the puerperium, *P00-P96* Certain conditions originating in the perinatal period, *Q00-Q99* Congenital malformations, deformations and chromosomal abnormalities, *R00-R99* Symptoms, signs and abnormal clinical and laboratory findings, not elsewhere classified, *S00-T98* Injury, poisoning and certain other consequences of external causes, *Z00-Z99* Factors influencing health status and contact with health services

Before matching, statistically significant differences (SMD > 0.2) existed between case and control groups for almost all variables except insurance, region and receiving surgery. Matching mitigated these imbalances through both conventional PSM and risk set matching. However, compared to the risk set matching, conventional PSM achieved greater balance between case and control groups.

### LOS and charges

The results for LOS and charges in the different models are presented in Table 2. In model 1, the attributable mean LOS and charges for those with and without BSI were 25.06 days and US$22041.73, respectively. The results in model 2 and 3 were both smaller than the baseline (model 1). Attributable LOS was less in the risk set matching model (16.86 days) than in the conventional propensity score matching model (21.27 days). Likewise, charges attributable to BSI were less in model 3 ($15,909.21) than model 2 ($18,549.47). Regrading to the component of additional hospital charges in the three models revealed that western medicine accounted for the largest proportion of charges, followed by laboratory and treatment (without surgery) fee.

**Table 2:** Comparison of outcomes with different models, 2017

Outcome	BSI	Model 1		Model 2		Model 3	
		Mean	Difference	Mean	Difference	Mean	Difference
LOS, days	34.45	9.39	25.06^*^	13.18	21.27^*^	17.59	16.86^*^
Total charges, USD	27,102.16	5060.43	22,041.73^*^	8552.69	18,549.47^*^	11,192.94	15,909.21^*^
Treatment, USD							
Treatment (no-surgery)	3119.80	357.47	2762.32^*^	744.72	2375.08^*^	1140.00	1979.80^*^
Surgery	551.73	426.62	125.11^*^	564.72	−12.99	620.70	−68.79
Nursing	223.48	37.14	186.34^*^	63.54	159.94^*^	96.03	127.45^*^
Anesthesia	82.26	54.21	28.05^*^	70.68	11.58	69.87	12.39^*^
Laboratory & examination, USD							
Laboratory	3713.34	543.02	3170.31^*^	1111.61	2601.72^*^	1327.96	2385.37^*^
Radiology	610.32	242.84	367.48^*^	295.55	314.76^*^	353.69	256.62^*^
Medicine & material, USD							
Western medicine	13,322.52	1573.54	11,748.98^*^	3477.33	9845.19^*^	4747.36	8575.16^*^
Medical material	4629.44	1961.10	2668.34^*^	2404.98	2224.46^*^	2845.10	1784.34^*^
Blood	976.72	221.17	755.54^*^	298.85	680.86^*^	547.96	428.75^*^
Chinese medicine	62.44	70.36	−7.91	44.04	18.41	60.69	1.75
Bed & meal, USD	696.74	149.77	546.97^*^	215.15	481.59^*^	328.49	368.25^*^
Other, USD	47.94	14.63	33.31^*^	20.62	27.32^*^	25.78	22.16^*^

Model 1, no matching; Model 2, conventional propensity score matching; Model 3, risk set matching. *BSI* Bloodstream infection, *LOS* Length of stay. T test was applied for mean cost and LOS. *P-value< 0.05

Results were similar for the sensitivity analysis that defined infection time as 2 days before the original infection date. For conventional matching (model 2), the attributable LOS due to BSI was 21.65 days, and the attributable costs were $19,530.87. These estimates were also much larger than estimates using risk-set matching (model 3), with additional 15.97 days in LOS and $15,914.98 in hospital charges due to BSI.

### Microorganisms and antimicrobial resistance bacteria

The isolated microorganisms and multi-drug resistance microorganisms of BSI are summarized in Table 3. Six hundred fourteen microorganisms were cultured from 557 episodes of BSI, most of the isolated pathogens (82.76%) were monomicrobial. The proportion of gram-negative versus gram-positive bacteria was 56.18% versus 31.92%. *Escherichia coli* was the most common bacteria (106, 17.26%). Among the 109 multi-drug resistant bacteria, carbapenem-resistant *Acinetobacter baumannii* (CRAB) was the most common (42, 38.53%).

**Table 3:** Isolated microorganisms and multi-drug resistance microorganisms involved in 557 episodes of bloodstream infection (BSI)

Microorganism	No. (%) of isolations	No. (%) of episodes with monomicrobial	No. (%) of isolations of specific antimicrobial-resistant pathogen
**Gram-negative bacteria**	**345 (56.18)**	**290 (84.06)**	**90 (26.09)**
*Escherichia coli*	106 (30.72)	90 (84.91)	3 (2.83)^a^
*Klebsiella pneumoniae*	88 (25.51)	80 (90.91)	30 (34.09)^a^
*Acinetobacter baumannii*	52 (15.07)	41 (78.85)	42 (80.77)^b^
*Pseudomonas aeruginosa*	24 (6.96)	19 (79.17)	13 (54.17)^c^
*Enterobacter cloacae*	23 (6.67)	15 (65.22)	2 (8.70)^a^
Other	52 (15.07)	45 (86.54)	–
**Gram-positive bacteria**	**196 (31.92)**	**162 (82.65)**	**19 (9.74)**
*Enterococcus faecium*	42 (21.43)	33 (78.57)	1 (2.38)^d^
*Staphylococcus epidermidis*	26 (13.27)	25 (96.15)	14 (53.85)^e^
*Staphylococcus aureus*	23 (11.73)	23 (100)	4 (17.39)^f^
*Staphylococcus hominis*	16 (8.16)	12 (75)	–
*Enterococcus faecalis*	13 (6.63)	8 (61.54)	–
Other	76 (38.78)	61 (80.26)	–
**Fungus**	**68 (11.07)**	**51 (75)**	–
*Candida species*	65 (95.59)	48 (73.85)	–
Other	3 (4.41)	3 (100)	–
**Anaerobic bacteria**	**5 (0.81)**	**5 (100)**	–
**Total**	**614 (100)**	**508 (82.74)**	**109 (17.75)**

^a^carbapenem-resistant *Enterobacteriaceae*

^b^carbapenem-resistant *Acinetobacter baumannii*

^c^carbapenem-resistant *Pseudomonas aeruginosa*

^d^vancomycin-resistant *Enterococcus faecium*

^e^methicillin-resistant *Staphylococcus epidermidis*

^f^methicillin-resistant *Staphylococcus aureus*

## Discussion

To our knowledge, this is the first analysis of LOS and charges attributable to HAIs in China to address confounding and time-dependent bias. Few international databases related to HAI collect infection time data [11]. Even studies that include infection time usually lack information about time-varying covariates, such as catheter insertion and removal time [12]. In this study, the real-time surveillance system collected and updated all HAI-related variables daily, allowing for time-dependent analysis based on dynamic information.

Baseline differences in age, sex, Charlson score, ICU admission, and procedures with device (central line catheter, urinary catheter, ventilator) between BSI and non-BSI patients were similar to a Belgian national surveillance study, however, the BSI patients’ average age (57.02 VS 66.9), Charlson comorbidity index (2.20 VS 3.10), and proportion of ICU admissions (16.2% VS 21.9%) were lower than the Belgian study [4].

The most common BSI pathogen was *Escherichia coli* (17.26%), consistent with results from the European antimicrobial resistance surveillance study [21]. However, other studies also report high BSI incidence due to *Staphylococcus aureus*, which was less common in our study (3.75%) [22, 23]*.*

Our results indicate LOS and charges attributable to HAI ranging from 16.86 to 25.06 days and $15,909.21 to $22,041.73, respectively. A systematic review found that LOS attributable to BSI ranged from 1.2–26.4 days, hence, our results were at the higher end relative to studies included in the review [6]. The published studies showed that attributable BSI cost ranged from $1430 (Brazil) [24] to $95,440 (US) [25]. Compared to the international study, the attributable BSI charges in our study is relatively low. However, it’s much higher than the study about CLABSIs in China (US$3528.6) [15].

Excess LOS is the main driver of hospital charges attributable to HAI. LOS and charges can be reduced through two potential approaches: BSI prevention and reductions in LOS for patients with BSI. A meta-analysis found that 57.78% of CLABSIs are preventable through intervention, such as insertion and maintenance bundle, which includes maximal barrier precautions, site selection, and better aseptic technique [26].*.* Some studies indicate long-term sustainability of zero CLABSIs is possible through high compliance with the bundles and multidisciplinary team interventions [27]. Some novel interventions introduced to shorten the LOS include real-time active alerts of positive blood cultures and antimicrobial stewardship intervention in patients with gram-negative bacteremia [28].

To reduce time-dependent bias, we applied risk-set matching with time-varying Cox regression [12], taking into consideration infection time as well as time-varying covariates (central venous catheter, urinary catheter, ventilator). The magnitude of time-dependent bias may vary depending on a series of factors, including infection rate, discharge rate, and confounders [11]. Sensitivity analysis was used in this study to assess the impact of unobserved confounding and determine to what degree unobserved variable might explain the results [12]. Findings from this study indicate future HAI surveillance should consider infection time (or specimen culture time). Time-varying procedures and treatments, such as device operation, surgery, and antibiotic administration should be recorded dynamically if possible.

This study was conducted from the hospital perspective, only calculating in-hospital medical charges. Owing to the non-profit nature of public hospitals in China, the charges were almost equal to the costs. In 2017, revenue and expenditure data from 44 top tertiary hospitals indicated that the charge-to-cost ratio was around 1.04 in China, compared to the value 2.0 in the U.S. [29, 30]. Hence, public hospitals in China may have less motivation to actively prevent HAI and reduce patients’ cost than in other settings.

Moreover, less than 30% of patients had public insurance in this study. Public insurance is not reimbursable outside patients’ city of permanent residence, hence, 7660 (6.46%) non-local patients with public insurance were not eligible to use their insurance. Thus, much of the HAI economic burden was borne by patients’ out-of-pocket instead of the hospital, perhaps providing less incentive to prevent HAIs [31]. However, actual economic savings include not only “cash savings” (variable cost), but also depend on the fixed costs, including buildings and equipment. While preventing HAI may not lead to “cash savings”, it could free up the limited medical resources for other revenue-generating activities [9].

In addition, payment reform was launched in Mainland China in 2017, gradually changing medical insurance payment methods from fee-for service (FFS) to Diagnosis Related Groups (DRGs) [32]. The prospective payment method will shift more of the economic burden of HAI to hospitals. Therefore, hospital administrators may prioritize reducing LOS and costs attributable to HAIs going forward.

This study has several limitations. First, single center studies limit generalization. The economic level and healthcare service prices vary in different provinces in China. Second, selection bias cannot be avoided in observational cohort studies with matched samples. We used logistic regression and Cox regression to create propensity scores and conduct the matching, since the confounders included in the study would change the results of matching. Third, our study evaluated cost only from the hospital perspective, neither including indirect cost from societal perspectives, nor collected patients’ cost after hospital discharge.

## Conclusions

In summary, ignoring time of infection will overestimate length of hospital stay and hospital charges attributable to BSI. This study can serve as a useful reference for future cost-effectiveness analyses of BSI interventions.

## Data Availability

The datasets generated during the current study are not publicly available, to avoid disclosure of the individual privacy of the patients. However, they are available from the corresponding author (LIU Yunxi: liuyunxi301@qq.com) on reasonable request.
